# Supplementary material for: Multistage protective anti-CelTOS monoclonal antibodies with cross-species sterile protection against malaria
Source: Nat Commun. 2024 Aug 29;15:7487. doi: 10.1038/s41467-024-51701-2 (PMC11362571; doi:10.1038/s41467-024-51701-2)
Supplement: Supplementary file 1 — Supplementary Information [file 41467_2024_51701_MOESM1_ESM.pdf]

Supplementary information for:

Multistage protective anti-CelTOS monoclonal antibodies with cross-species sterile protection against malaria

Wai Kwan Tang[1], Nichole D. Salinas[1], Surendra Kumar Kolli[2], Shulin Xu[2], Darya Urusova[3], Hirdesh Kumar[1], John R Jimah[3,4], Pradeep Annamalai Subramani[2], Madison M. Ogbondah[2], Samantha J. Barnes[2], John H. Adams[2], Niraj H. Tolia[1]*

[1]Host–Pathogen Interactions and Structural Vaccinology Section, Laboratory of Malaria Immunology and Vaccinology, National Institute of Allergy and Infectious Diseases, National Institutes of Health, Bethesda, MD, USA

[2] Center of Global Health and Interdisciplinary Research, College of Public Health, University of South Florida, Tampa, FL, USA

[3]Department of Molecular Microbiology, Washington University School of Medicine, St. Louis, MO, USA

[4]Current address: Department of Molecular Biology, Princeton University, Princeton, NJ, USA

*Corresponding author: niraj.tolia@nih.gov

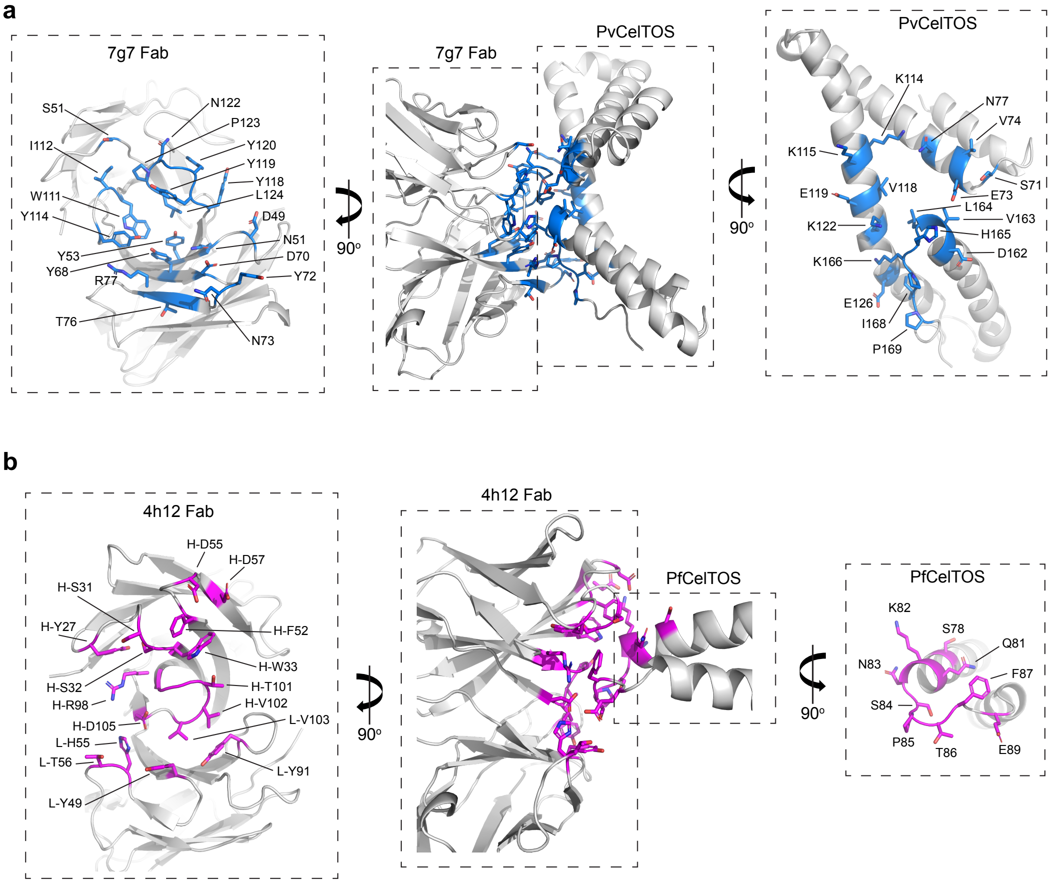

Supplementary Fig 1: Structural definition of antibody binding epitopes in CelTOS. Orthogonal detailed view of binding epitopes on **a** 7g7 and PvCelTOS and **b** 4h12 and PfCelTOS. The antigen-antibody complexes were shown in the middle panels. Residues in the binding epitopes were labeled and highlighted in corresponding color.

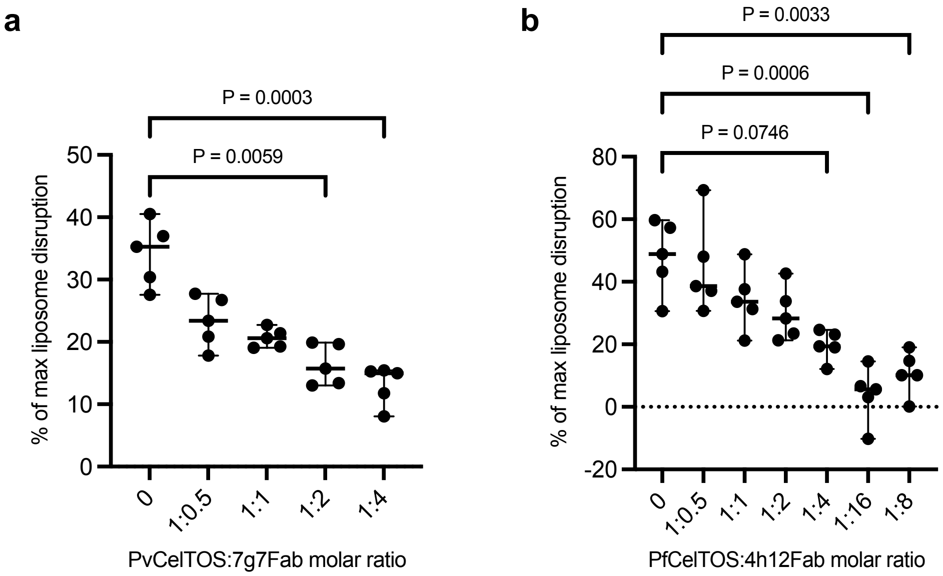

Supplementary Fig 2: Both 7g7 and 4h12 inhibited CelTOS-mediated membrane disruption. Concentration dependent inhibition of **a** 7g7 to PvCelTOS and **b** 4h12 to PfCelTOS in liposome disruption assays. The median with 95% CI and five replicates is shown. Significance was determined using a nonparametric Kruskal-Wallis test with Dunn's correction for multiple comparisons. The mean ± s.e.m from five replicates are shown.

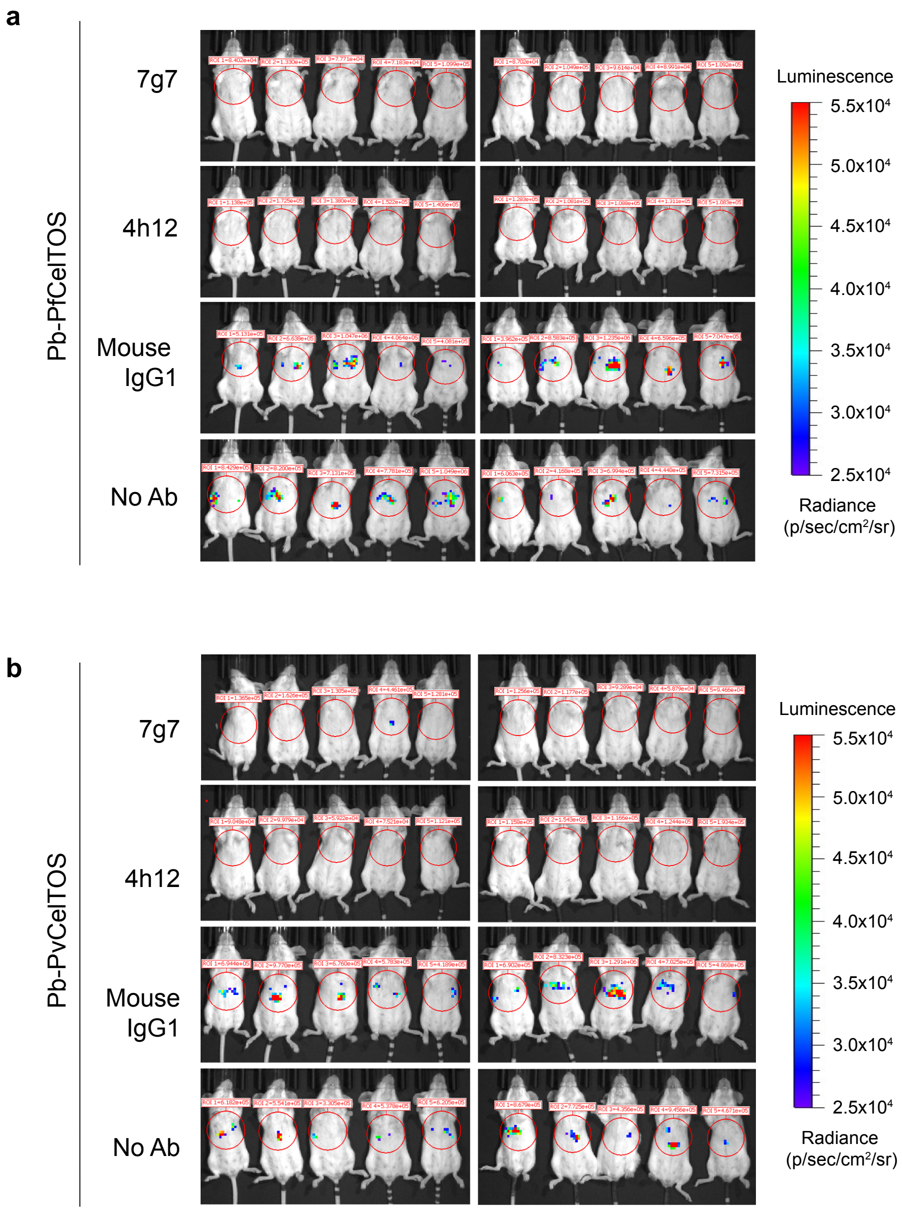

Supplementary Fig 3: The parasite liver loads in mice.
Two hundred GFP-luciferase expressing sporozoites of either **a** Pb-PfCelTOS or **b** Pb-PvCelTOS were injected to challenge mice immunized with different antibodies. Antibody isotype matched control group was done using anti-PvCSP VK247 antibody 2E10.E9 [38]. The parasite liver loads were measured at 44 hr post infection. The luciferase activities of parasites were measured using IVIS Lumina II Imaging System and the value of RLU of each mouse was reported.

PvCelTOS MNKVNRVSIICAFLALFCFVNVLSLRGKSGSTASSSLEGGSEFSERIGNSLSSFLSESA**S**L**EV**IG**N**ELAD 70

PvCelTOS NIANEIVSSLQKDSASFLQSGFDVKTQLKATA**KK**VL**VE**AL**K**AAL**E**PTEKIVASTIKPPRVSEDAYFLLGP 140

PvCelTOS VVKTLFNKV**EDVLHKPIP**DTIWEYESKGSLEEEEAEDEFSDELLD 185

Amino acid	Position	Polymorphic change
**G**	10	S
**S**	20	N
**V**	118	L/M
**K**	178	T
**G**	179	R/D
**L**	181	R

Supplementary Fig 4: Non-synonymous mutations in PvCelTOS.
Data is from Bitencourt Chaves [39] and Mehrizi [40] based on analysis of 217 samples from Brazil, Iran, Thailand, Columbia, Mexico and Peru, in compare with PvCelTOS Sal-1 sequence. Polymorphic residues were highlighted in cyan. Epitope residues of 7g7 were in blue. Polymorphic change of the residues was listed.

```
PfCelTOS   MNALRRLPVICSFLVFLVFSNVLCFRGNNGHNSSSSLYNGSQFIEQLNNSFTSAFLESQSMNKIGDDLAE   70

PfCelTOS   TISNELVSVLQKNSPTFLESSFDIKSEVKKHAKSMLKELIKVGLPSFENLVAENVKPPKVDPATYGIIVP   140

PfCelTOS   VLTSLFNKVETAVGAKVSDEIWNYNSPDVSESEESLSDDFFD
```

Amino acid	Position	Polymorphic change	MAF globle
**A**	3	T/V	0.000/0.000
**L**	17	F	0.000
**G**	30	R	0.000
**S**	53	L/T	0.000/0.001
**K**	100	N/R	0.143/0.192
**H**	101	N	0.024
**L**	106	F	0.051
**K**	107	N	0.021
**E**	108	Q	0.003
**V**	112	A	0.001
**S**	116	P	0.017
**F**	117	L/S/V	0.000/0.000/0.127
**N**	119	H/K/S	0.004/0.196/0.009
**L**	120	V	0.251
**V**	121	I	0.171
**E**	123	A/Q	0.017/0.047
**V**	125	I	0.002
**V**	141	L	0.022
**T**	151	S	0.017
**A**	155	T/V	0.048/0.313
**K**	156	N/R	0.039/0.043
**N**	163	D/K/H	0.001/0.018/0.031

Supplementary Fig 5: Non-synonymous mutations in PfCelTOS.
Data is from MalariaGEN *Plasmodium falciparum* Community Project Pf4 dataset, based on analysis of 3,488 samples from 23 countries (https://www.malariagen.net/parasite/p-falciparum-community-project), in compare with PfCelTOS 3D7 sequence. Polymorphic residues were highlighted in cyan. Epitope residues of 4h12 were in magenta. Polymorphic change and global minor allele frequency, MAF, of the residues was listed.

Supplementary Table 1. Data collection, phasing and refinement statistics

	PfCelTOS-4h12	PvCelTOS-7g7
**Data collection**		
Space group	P1	P1
Cell dimensions		
a, *b*, *c* (Å)	59.33, 59.29, 153.52	182.89 78.62 47.32
α, β, γ (°)	82.18, 82.04, 72.27	90.03 95.37 89.88
Resolution (Å)	19.86-3.52 (3.64-3.52)	48.00-3.20 (3.31-3.20)
R_{meas}	12.80 (55.10)	4.40 (55.80)
I/σ	6.28 (1.72)	10.31 (2.35)
$CC_{1/2}$	99.10 (82.50)	99.90 (91.50)
Completeness (%)	97.70 (97.70)	90.56 (92.99)
Redundancy	1.98 (1.97)	2.11 (2.16)
Beamline	ALS 4.2.2	APS SER-CAT
No. of complex/ASU	4	4
PDB	8UKH	8ULF
**Refinement**		
Resolution (Å)	19.86 – 3.52	48.00-3.20
No. reflections	23,939	39,227 (4,047)
R_{work} / R_{free}	26.76/29.35	27.52/29.54
No. atoms		
Protein	14,752	16,908
Water	11	---
B-factors		
Protein	51.76	130.34
Water	11.58	---
R.m.s. deviations		
Bond lengths (Å)	0.003	0.003
Bond angles (°)	0.65	0.62
Validation		
MolProbity score	1.50	1.71
Clashscore	4.55	8.27
Poor rotamers (%)	0	0
Ramachandran plot		
Favored (%)	96.08	96.15
Allowed (%)	3.92	3.85
Disallowed (%)	0	0

Highest resolution shell is shown in parenthesis.

Supplementary Table 2: Table of interacting residues for PvCelTOS and 7g7 Fab complex. CDR indicates complementarity-determining region.

PvCelTOS residues	Interaction	Interface with 7g7
S71		Light chain
E73		Light chain
E73		Heavy chain
V74		Light chain
N77		Heavy chain
K114		Heavy chain
K115		Heavy chain
V118		Heavy chain
E119		Heavy chain
K122		Heavy chain
E126		Heavy chain
E161		Heavy chain
D162	H-bond & Salt bridge	Heavy chain
D162	H-bond	Light chain
V163		Heavy chain
V163		Light chain
L164		Heavy chain
H165	H-bond	Heavy chain
H165		Light chain
K166		Heavy chain
P167	H-bond	Heavy chain
I168		Heavy chain
P169		Heavy chain

7g7 residues	CDR
D49	H1
N51	H1
Y53	H1
Y68	H2
I69	H2
D70	H2
Y72	H2
N73	H2
G75	H2
T76	H2
R77	H2
Y118	H3
Y119	H3
Y120	H3
G121	H3
N122	H3
P123	H3
L124	H3
S51	L1
W111	L3
I112	L3
Y114	L3

Supplementary Table 3: Table of interacting residues for PfCelTOS and 4h12 Fab complex. CDR indicates complementarity-determining region.

PfCelTOS residues	Interaction	Interface with 4h12
S78		Heavy chain
Q81		Heavy chain
K82	H-bond & Salt bridge	Heavy chain
N83		Heavy chain
S84	H-bond	Heavy chain
P85	H-bond	Heavy chain
P85		Light chain
T86		Heavy chain
T86		Light chain
F87	H-bond	Heavy chain
E89		Heavy chain
E89	Salt bridge	Light chain

4h12 residues	CDR
Y27	--
A28	--
S31	H1
S32	H1
W33	H1
F52	H2
D55	H2
D57	H2
R98	H3
G99	H3
G100	H3
T101	H3
V102	H3
V103	H3
D105	H3
Y49	L2
W50	L2
H55	L2
T56	L2
Y91	L3

Supplementary Table 4 Binding affinities of mAbs towards CelTOS using BLI. mAbs were immobilized onto anti-mouse IgG Fc capture (AMC) biosensors. The data was fitted using a 1:1 binding ratio. The kinetics was analyzed using Octet Data Analysis HT 12 software. Each biological replicate was the average of three repeats, and the mean was the average of the three biological replicates.

			K_D (x 10^{-9} ± SEM M)	k_a (x 10^4 ± SEM 1/Ms)	k_{dis} (x 10^{-4} ± SEM 1/s)	N
	7g7 & PvCelTOS	Biological replicate 1	58.50 ± 6.00	6.23 ± 0.32	36.77 ± 4.60	3
		Biological replicate 2	39.50 ± 2.57	5.70 ± 0.23	22.67 ± 2.27	3
		Biological replicate 3	54.20 ± 6.94	6.13 ± 0.58	33.20 ± 5.36	3
		Mean	50.73 ± 5.75	6.02 ± 0.16	30.88 ± 4.23	
	4h12 PfCelTOS	Biological replicate 1	65.93 ± 0.32	8.63 ± 0.24	56.87 ± 1.58	3
		Biological replicate 2	67.97 ± 6.52	8.97 ± 0.23	60.87 ± 4.81	3
		Biological replicate 3	62.30 ± 6.44	8.47 ± 0.48	52.10 ± 2.62	3
		Mean	65.40 ± 1.66	8.69 ± 0.15	56.61 ± 2.54	

			K_D (x 10^{-6} ± SEM M)	k_a (x 10^4 ± SEM 1/Ms)	k_{dis} (x 10^{-4} ± SEM 1/s)	N
Cross-species	7g7 & PfCelTOS	Biological replicate 1	0.47 ± 0.018	0.59 ± 0.02	27.87 ± 1.48	3
		Biological replicate 2	0.48 ± 0.059	0.60 ± 0.01	29.27 ± 3.41	3
		Biological replicate 3	0.50 ± 0.026	0.56 ± 0.02	28.10 ± 2.10	3
		Mean	0.48 ± 0.009	0.58 ± 0.01	28.41 ± 0.43	
	4h12 PvCelTOS	Biological replicate 1	41.62 ± 7.66	0.11 ± 0.0.2	410.7 ± 23.60	3
		Biological replicate 2	57.48 ± 8.60	0.09 ± 0.006	511.20 ± 47.35	3
		Biological replicate 3	56.3 ± 12.21	0.11 ± 0.02	549.50 ± 72.68	3
		Mean	51.81 ± 5.11	0.10 ± 0.006	490.50 ± 41.39	

Supplementary Table 5 Amino acid sequences of the variable regions of anti-CelTOS antibodies.

Antibody	Sequence
7g7<br>Heavy chain	EIQLQQSGPELVKPGASVKVSCKASGYSFTDYNMYWVKQSHGKSLEWIGYIDPYN<br>GGTRYNQKFRDKATLTVDKSSSTAFMHLNSLTSEDSAVYYCARGYYYGNPLHFDV<br>WGAGTTVTVSS
7g7<br>Light chain	QIVLTQSPAIMSASPGEKVTMTCSASSSVSYIHWYQQKSGTSPKRWIYDTSK<br>LASGVPARFSGSGSGTSYSLTISSMEAEDAATYYCQQWISYPATFGAGTKLE<br>LK
4h12<br>Heavy chain	QVQLQQSGPELVKPGASVKISCKASGYALSSSWLNWVKQRPGQGLEWIGRIFPGD<br>GDTNYNGKFKGKATLTADKSSSTAYLQLSSLTSVDSAVYFCARGGTVVFDYWGQG<br>TTLTVSS
4h12<br>Light chain	DIVMTQSHKFMSTSVGDRVNITCKASQDVGIAVAWYQQRPGQSPKLLIYWASKRH<br>TGVHDRFTGTGSGTDFTLTISTVQSEDLADYFCQQYSNSLTFGAGTTLELS
