# Supplementary material for: Multistage protective anti-CelTOS monoclonal antibodies with cross-species sterile protection against malaria
Source: Nat Commun. 2024 Aug 29;15:7487. doi: 10.1038/s41467-024-51701-2 (PMC11362571; doi:10.1038/s41467-024-51701-2)
Supplement: Supplementary file 3 — Reporting Summary [file 41467_2024_51701_MOESM3_ESM.pdf]

nature portfolio

Corresponding author(s): Niraj Tolia

Last updated by author(s): Jun 21, 2024

Reporting Summary

Nature Portfolio wishes to improve the reproducibility of the work that we publish. This form provides structure for consistency and transparency in reporting. For further information on Nature Portfolio policies, see our Editorial Policies and the Editorial Policy Checklist.

Statistics

For all statistical analyses, confirm that the following items are present in the figure legend, table legend, main text, or Methods section.

n/a	Confirmed	
☐	☒	The exact sample size (*n*) for each experimental group/condition, given as a discrete number and unit of measurement
☐	☒	A statement on whether measurements were taken from distinct samples or whether the same sample was measured repeatedly
☐	☒	The statistical test(s) used AND whether they are one- or two-sided<br>*Only common tests should be described solely by name; describe more complex techniques in the Methods section.*
☒	☐	A description of all covariates tested
☐	☒	A description of any assumptions or corrections, such as tests of normality and adjustment for multiple comparisons
☐	☒	A full description of the statistical parameters including central tendency (e.g. means) or other basic estimates (e.g. regression coefficient) AND variation (e.g. standard deviation) or associated estimates of uncertainty (e.g. confidence intervals)
☐	☒	For null hypothesis testing, the test statistic (e.g. *F*, *t*, *r*) with confidence intervals, effect sizes, degrees of freedom and *P* value noted<br>*Give P values as exact values whenever suitable.*
☒	☐	For Bayesian analysis, information on the choice of priors and Markov chain Monte Carlo settings
☒	☐	For hierarchical and complex designs, identification of the appropriate level for tests and full reporting of outcomes
☒	☐	Estimates of effect sizes (e.g. Cohen's *d*, Pearson's *r*), indicating how they were calculated

Our web collection on statistics for biologists contains articles on many of the points above.

Software and code

Policy information about availability of computer code

Data collection	Octet BLI Discovery 12.2.2.20 software was used to collect the bio-layer interferometry data. Gen5 3.08.01 software was used to collect the ELISA data. Zen blue software v3.6 was used for imaging in sporozoite immunofluorescence assay. IVIS Lumina II Imaging System was used to record luciferase activity in sporozoite challenge study.
Data analysis	-GraphPad Prism 9.3.1<br>-Octet Analysis Studio 12.2.2.26 software<br>-XDS Package, Phenix 1.20.1, Coot 0.9.8.82 and PyMol curated by SBGrid

For manuscripts utilizing custom algorithms or software that are central to the research but not yet described in published literature, software must be made available to editors and reviewers. We strongly encourage code deposition in a community repository (e.g. GitHub). See the Nature Portfolio guidelines for submitting code & software for further information.

Data

Policy information about availability of data

All manuscripts must include a data availability statement. This statement should provide the following information, where applicable:

- Accession codes, unique identifiers, or web links for publicly available datasets
- A description of any restrictions on data availability
- For clinical datasets or third party data, please ensure that the statement adheres to our policy

All data generated or analyzed during this study are included in this published article, source data file and supplementary information files. Atomic coordinates and structure factors have been deposited in the Protein Data Bank with PDB accession number 8ULF for PvCelTOS-7g7 and 8UKH for PfCelTOS-4h12 structures.

Research involving human participants, their data, or biological material

Policy information about studies with human participants or human data. See also policy information about sex, gender (identity/presentation), and sexual orientation and race, ethnicity and racism.

Reporting on sex and gender	Not applicable
Reporting on race, ethnicity, or other socially relevant groupings	Not applicable
Population characteristics	Not applicable
Recruitment	Not applicable
Ethics oversight	Not applicable

Note that full information on the approval of the study protocol must also be provided in the manuscript.

Field-specific reporting

Please select the one below that is the best fit for your research. If you are not sure, read the appropriate sections before making your selection.

[x] Life sciences [ ] Behavioural & social sciences [ ] Ecological, evolutionary & environmental sciences

For a reference copy of the document with all sections, see nature.com/documents/nr-reporting-summary-flat.pdf

Life sciences study design

All studies must disclose on these points even when the disclosure is negative.

Sample size	Sample sizes were chosen based on animal availability and the ability to see trends in the event of one or two outlier animals. The groups sizes are sufficient to support the primary conclusion of the manuscript with statistically significant differences.
Data exclusions	No data points were excluded from statistical analysis
Replication	In vitro assays were independently replicated at lease twice to ensure reproducibility, and all replication attempts were successful. Sporozoite challenge study was repeated successfully twice and the results from the latest attempt was reported.
Randomization	Animals were randomly assigned to receive the indicated treatments.
Blinding	Groups were not blinded for the animals studies.

Reporting for specific materials, systems and methods

We require information from authors about some types of materials, experimental systems and methods used in many studies. Here, indicate whether each material, system or method listed is relevant to your study. If you are not sure if a list item applies to your research, read the appropriate section before selecting a response.

Materials & experimental systems

n/a	Involved in the study
☐	☒ Antibodies
☐	☒ Eukaryotic cell lines
☒	☐ Palaeontology and archaeology
☐	☒ Animals and other organisms
☒	☐ Clinical data
☒	☐ Dual use research of concern
☒	☐ Plants

Methods

n/a	Involved in the study
☒	☐ ChIP-seq
☒	☐ Flow cytometry
☒	☐ MRI-based neuroimaging

Antibodies

Antibodies used	-mAb 7g7 and 4h12 were purified from hybridoma cells.<br>-Anti-mouse HRP-conjugated secondary antibody (1:5000) from Jackson ImmunoResearch (catalog no.: 112-035-071) was used for ELISA<br>-Anti-mouse antibody conjugated to Alexa Fluor Plus 488 from Thermo Fisher Scientific for sporozoite immunofluorescene assay
Validation	-mAb 7g7 and 4h12 were validated in this study by hybridoma sequencing and De novo sequencing.<br>- The anti-mouse HRP-conjugated secondary antibody was validated in numerous previous scientific publications and by the manufacturer.<br>-The anti-mouse antibody conjugated to Alexa Fluor Plus 488 was validated in numerous previous scientific publications and by the manufacturer.

Eukaryotic cell lines

Policy information about cell lines and Sex and Gender in Research

Cell line source(s)	Expi293F™ cells were purchased from Thermo Fisher Scientific (cat#A14527).
Authentication	Expi293F™ cells were authenticated for Viability and Mycoplasma (Mycoplasma qPCR Assay) by Thermo Fisher Scientific. Expi293F™ cells were more than 90 % viable and negative for Mycoplasma.
Mycoplasma contamination	The cells were not tested for Mycoplasma contamination. No previous case of contamination was ever detected in our laboratory.
Commonly misidentified lines (See ICLAC register)	No commonly misidentified lines were used.

Animals and other research organisms

Policy information about studies involving animals; ARRIVE guidelines recommended for reporting animal research, and Sex and Gender in Research

Laboratory animals	Five- to six-week old female BALB/c mice from Taconic Biosciences for generation of mouse monoclonal antibodies against CelTOS. Six- to eight-week old female Swiss Webster ND4 mice (Inotiv, West Lafayette, IN) were used in sporozoite production and six- to eight-week old female female BALB/c mice (Inotiv, West Lafayette, IN) were used in sporozoite challenge studies.
Wild animals	The study did not involve wild animals.
Reporting on sex	Sex was not considered in the study design.
Field-collected samples	The study did not use field-collected samples
Ethics oversight	Mouse immunizations to generate mAbs were performed in adherence to the regulations of the Institutional Animal Care and Use Committee (IACUC) at Washington University in St. Louis under Animal Welfare Assurance #D1600245. Washington University in St. Louis has full accreditation from the Association for Assessment and Accreditation of Laboratory Animal Care International (AAALAC). All challenge studies were performed in adherence to the regulations of the Institutional Animal Care and Use Committee (IACUC) at the University of South Florida.

Note that full information on the approval of the study protocol must also be provided in the manuscript.

Plants

Seed stocks	Not applicable.
Novel plant genotypes	Not applicable
Authentication	Not applicable
